# A Proposal of Implementation of Sitting Posture Monitoring System for Wheelchair Utilizing Machine Learning Methods

**DOI:** 10.3390/s21196349

**Published:** 2021-09-23

**Authors:** Jawad Ahmad, Johan Sidén, Henrik Andersson

**Affiliations:** Department of Electronics Design, Mid Sweden University, 851 70 Sundsvall, Sweden; johan.siden@miun.se (J.S.); henrik.andersson@miun.se (H.A.)

**Keywords:** pressure sensor, pressure mapping, sitting posture recognition, wheelchair seat cushion, machine learning algorithms

## Abstract

This paper presents a posture recognition system aimed at detecting sitting postures of a wheelchair user. The main goals of the proposed system are to identify and inform irregular and improper posture to prevent sitting-related health issues such as pressure ulcers, with the potential that it could also be used for individuals without mobility issues. In the proposed monitoring system, an array of 16 screen printed pressure sensor units was employed to obtain pressure data, which are sampled and processed in real-time using read-out electronics. The posture recognition was performed for four sitting positions: right-, left-, forward- and backward leaning based on k-nearest neighbors (k-NN), support vector machines (SVM), random forest (RF), decision tree (DT) and LightGBM machine learning algorithms. As a result, a posture classification accuracy of up to 99.03 percent can be achieved. Experimental studies illustrate that the system can provide real-time pressure distribution value in the form of a pressure map on a standard PC and also on a raspberry pi system equipped with a touchscreen monitor. The stored pressure distribution data can later be shared with healthcare professionals so that abnormalities in sitting patterns can be identified by employing a post-processing unit. The proposed system could be used for risk assessments related to pressure ulcers. It may be served as a benchmark by recording and identifying individuals’ sitting patterns and the possibility of being realized as a lightweight portable health monitoring device.

## 1. Introduction

Individuals who require wheelchairs may be suffering from physical and or sensory disabilities which hinder their daily activities. Not only might a large number of human beings in different parts of the world be subjected to developing a type of pressure ulcer due to maintaining a sedentary lifestyle, but this may also happen because of disorders associated with reduced mobility and its complications. The well-being condition of a wheelchair bound individual is affected by prolonged sitting time and sitting posture [1,2]. A continuous pressure over a period of time results in restricted blood flow and the limitation of nutrition and oxygen supply to skin tissues, which may lead to localized and deep tissue injury (DTI) [3,4]. Generally, immobility may come from a broad spectrum of causes. The reasons for the loss of movement can be categorized into intrapersonal factors, which are linked to psychological parameters such as extreme level of stress or anxiety, fear of getting injured and, post-traumatic stress disorder (PTSD), as well as due to physical modifications that may have emerged as a result of injuries including—but not limited to—cardiovascular, cognitive and musculoskeletal conditions, which expose many groups to the possibility of developing pressure ulcers [5,6].

The endurance of pressure varies from person to person; nonetheless, a number of research studies have found that a pressure value greater than arterial capillary pressure (32 mm Hg) may lead to occlusion in blood vessels [7,8]. Furthermore, those who are unable to have any sort of movement without the help of others, which may usually be due to spinal cord injuries, Parkinson’s and osteoporotic fracture, are at significantly higher risk of developing pressure ulcers [9,10,11]. Among the frequent users of wheelchairs, pressure ulcer most commonly appears within a certain part of the body such as ischial tuberosities, calcaneus and back of the knee [12]. However, due to inadequate mobility, moisture anomalies, friction and shear, it can also develop in other skin regions. Therefore, one can conclude that identifying the appropriate sitting posture in the first place is virtually the best approach that can be taken to limit the prolonged intensity of the pressure and, thus, the probability of developing an ulcer can be decreased.

There are many diverse methods that have been developed to identify the sitting behavior of persons and to reduce the pressure ulcer development risk. Some related works have developed studies on sitting posture recognition using force sensors and vibration motors. The small motors vibrate after a bad posture is detected [13,14]. Another study performs a similar method of posture selection but utilizes normal chairs [15]. A textile pressure sensor is proposed in [16] and a sitting posture detection uses gyroscope readings through mobile devices attached to human spinal points and incorporates decision tree algorithm [17]. Furthermore, other studies have proved the benefit of using machine learning algorithms such as k-NN and SVM, along with sensors to classify human activities and reach a classification performance of around 80% [18,19]. Lying posture determination through recognition of multitier body parts is carried out in [20] and the system recognizes six different lying positions base on three major body parts such as head, shoulder and hips. A camera-based posture recognition system uses ensemble convolutional neural networks (CNNs) in home environments to cope with sudden danger to elderly people is reported [21].

In this paper, we present an intuitive method of sitting posture recognition system using pressure sensors and machine learning algorithms. The system is built on a pressure sensor array embedded in a wheelchair seat cushion, read-out electronics and the classification machine learning algorithms on a standard PC and embedded computing systems (raspberry pi 3B and raspberry pi 4B). The machine learning algorithms are evaluated using a conventional computer and raspberry pi embedded platform to verify its functionality. We have realized that the proposed system has a high level of sitting posture classification accuracy up to 99.03%, which may help in identifying and deciding about the most suitable sitting posture at different times. The contributions of this paper are listed as follows:

- The paper includes a pressure sensor design employing large area electronics, based on piezoresistive effect, using conductive inks and flexible substrates.
- A read-out electronics system is designed to acquire the pressure data from pressure sensor using a microcontroller.
- The paper investigates the applicability of machine learning schemes in the proposed pressure sensing system. A detailed performance comparison is presented between kNN, SVM, random forest, decision tree and LightGBM schemes.
- The proposed system is demonstrated on an embedded platform to present a standalone pressure recognition system, which is capable of achieving same classification accuracy as compared to a standard personal computer (PC).
- A computational comparison is presented between an embedded system and a standard PC in order to evaluate the performance of the proposed system on different platforms.

The rest of the paper has the following organization: Section 2 describes the overall system configuration that includes hardware and data processing characteristics, Section 3 demonstrates the results of the experiments and Section 4 reports the standalone configuration of the proposed system. Section 5 presents an analysis of results and Section 6 defines possible future directions.

## 2. System Configuration

The sitting posture recognition system is based on five main stages: (a) design and fabrication of screen-printed pressure sensors, (b) design of read-out electronics, (c) data acquisition and data set compilation, (d) data processing and classification algorithms using scikit-learn machine learning (version 0.24.2) [22] and LightGBM (version 3.2.1) [23] on standard PC and (e) implementation of the same algorithms on an embedded platform, e.g., raspberry pi to compare results with standard PC. The implementation on an embedded platform is carried out to investigate its potential functionalities of being used as a fully portable monitoring system, which would then contribute to user’s comfort.

### 2.1. Design of Pressure Sensing Matrix

**Figure 1:**
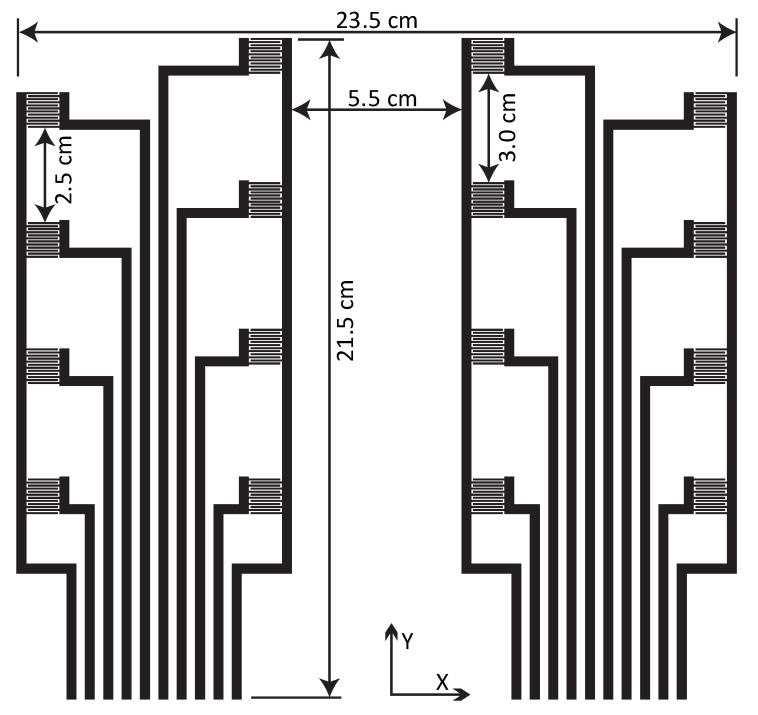
Area of the pressure sensor and placement of sensing elements.

The design of pressure sensing matrix is based on a general sitting area of a wheelchair seat cushion focusing on the ischial areas. The large area screen-printed sensor for wheelchair seat consists of 16 pressure sensing elements, covering an effective sitting area of 505 cm^2^. The horizontal and vertical dimensions for the sensor matrix are 23.5 cm × 21.5 cm as shown in Figure 1. The distance between sensing elements in sensor matrix is 5.5 cm in X direction, while 3.0 cm for central sensing elements and 2.5 cm for outer sensing elements in Y direction, respectively. These are common seating dimensions of a person with a normal physique for ischial areas. The sensor is usually placed inside or underneath the foam cushion. The assembly requires a very thin and flexible pressure sensor to be able to tolerate mechanical deformations due to user’s movements.

**Figure 2:**
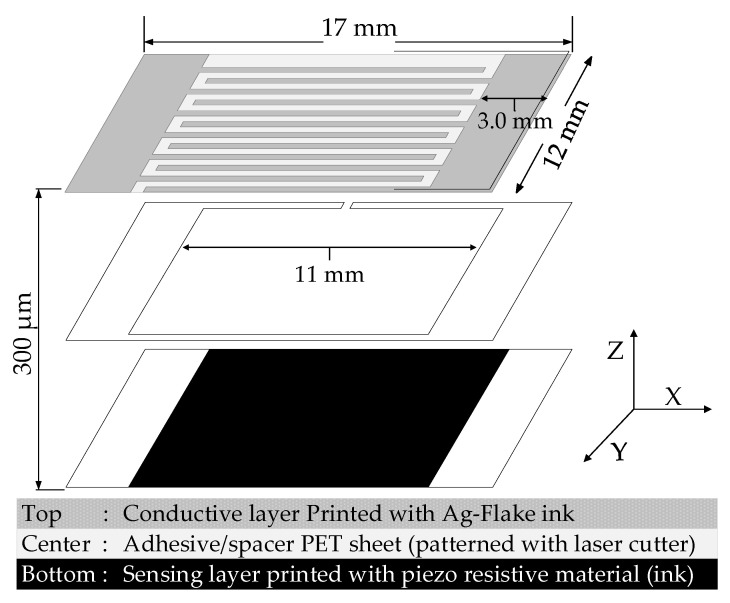
Exploded view structure of a sensing element.

Three PET sheets with a thickness of 100 µm, are employed to construct the proposed large area sensor, described as top, center and bottom sheets for this design. Top sheet is printed with interdigital patterns and interconnects with highly conductive Ag-ink and acts as a conductive layer. Bottom layer is printed with carbon filler based blended ink that has both resistive and piezoresistive properties. The center layer has openings that are patterned by a laser cutter, works as a separator between two layers. The center layer also functions as an adhesive bonding between top and bottom layers. The dimensions and configuration of sensing element are depicted in Figure 2.

The sensor matrix’s top layer is printed using an Ag flakes-based ink ECI-1036 by Engineered Conductive Materials (ECM), which has a stated sheet resistance 12–15 mΩ/sq/25 µm. The temperature curing is carried out for 5 min in a convection oven at 120 °C. On a PET sheet that is 100 µm thick, a wet layer of 10 µm thickness has been screen-printed using a 100–40 (100 threads per cm, 40 μm thread thickness) meshed screen employing a semi-automatic screen printer DY-2030P. Methyl Ethyl Ketone (MEK) is utilized to clean off dust and fat particles from PET sheets.

The sensing layer is printed using a blend of two inks, (CI-2062 by ECM) and (CI-7031 by ECM), which is a positive temperature coefficient carbon particle-based piezoresistive ink and a non-conducting ink, which offer controllable ranges of electrical resistance. These inks are combined with a 10% and 90% ratio, respectively, to achieve roughly 130–150 kΩ/sq/10 μm initial sheet resistance, which is empirically an ideal value (neither too resistive nor too conductive) for the scope of the design. The blending is carried out with a paddled stirrer at 100 rpm.

### 2.2. Read-Out Circuit Design for Sensor Matrix

**Figure 3:**
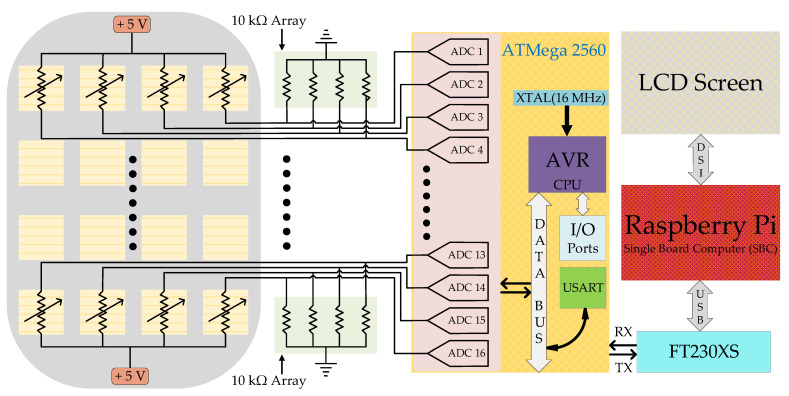
Explicit circuit model for standalone pressure sensing system.

The explicit system model is illustrated in Figure 3 shows the electrical circuitry. The pressure data from sensor matrix are acquired and sampled in the read-out electronics using an 8-bit Atmel ATmega-2560 microcontroller operating at 16 MHz. The microcontroller is programmed using the Arduino software package. When connected in a 5.0 V voltage divider configuration with 10 kΩ resistor, a single sensing element may draw a maximum of 0.56 mA current. The entire read-out circuit utilizes an average current of 100 mA. To get the output voltage (*V_out_*) of each sensor element utilizing a fixed resistor of 10 kΩ with 0.1% precision, the voltage divider configuration is utilized and the output voltage is calculated using Equation (1).

(1) $$V_{\mathrm{out}}=V_{\mathrm{in}}\;\times \;\frac{R_{10k}}{R_{10k}+R_{\mathrm{sensor}}}$$

**Figure 4:**
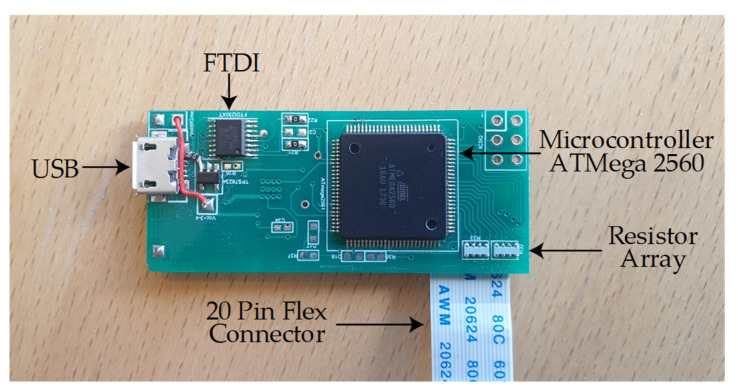
Read-out electronics on a 5 cm × 2 cm PCB.

The output voltage is transformed into digital values by utilizing 16 ADC channels of an ATmega-2560 microcontroller. The read-out electronics is situated on a 5 cm × 2 cm PCB as shown in Figure 4. The sensor matrix and read-out circuitry would easily fit within the seat cushion due to its compact form factor. The sensor matrix is connected to the PCB using a twenty-pin flex connector that has a 0.5 mm pitch.

### 2.3. Data Set Compilation

**Figure 5:**
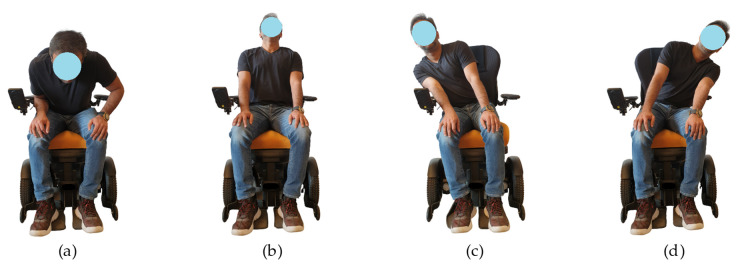
Collection of training data with different sitting positions: (**a**) Forward leaning; (**b**) Backward leaning; (**c**) Right leaning; (**d**) Left leaning.

To identify the specific sitting posture on a wheelchair and to label the collected measurements (hereafter referred to as samples) for use in posture classification schemes, we resort to a traditional taxonomy established by physiotherapists and physicians who have the expertise in this field. The following explains the data collecting procedure used in this study: 32 individuals took part in compilation of the dataset and they were instructed to hold each of four postures (a, b, c and d) demonstrated in Figure 5, for up to 30 s (average estimated time required to acquire a reliable sample). The stable values are then selected to make the training data set for four postures, i.e., left-, right-, forward- and backward lean called as class in the data set. Each individual completed one set per posture. Since the data are comprised of 16 sensors, a total of 4096 data points (256 observations × 16 sensors) is obtained. The average weight and weight range of the participants are presented in Table 1.

**Figure 6:**
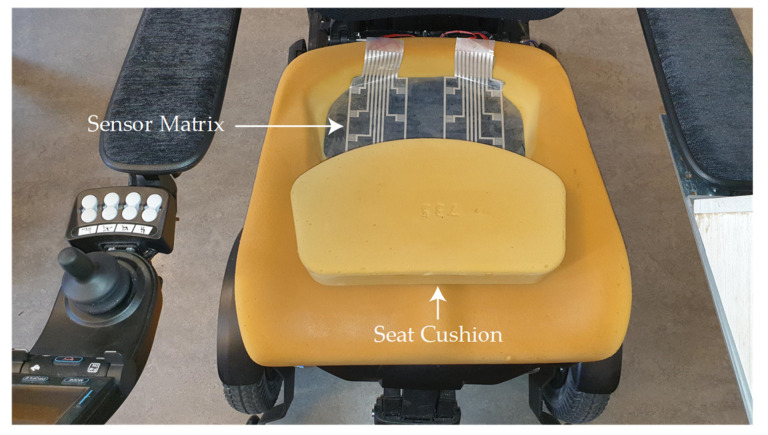
A foam-based seat cushion of a wheelchair with sensor matrix is placed inside the cushion.

The data sampling was performed at a temperature of 24 °C and relative humidity of 30–40% at the lean angles of ≈25° and ≈35° degrees. Participants are 38 ± 13 years in age and 175 ± 18 cm in height. The pressure points data for sitting and leaning positions from volunteers have been collected through a MATLAB^®^ GUI and that GUI simplifies the sample acquisition process (acquiring a sample at a specific time). The seat cushion of the wheelchair along with the placement of the sensor matrix are shown in Figure 6.

### 2.4. K-Nearest Neighbor (k-NN)

K-nearest neighbor is a supervised learning algorithm where the result of a new instance query is classified based on the majority of k-nearest neighbor categories [24]. It is one of the most popular algorithms for pattern recognition [25]. The purpose of this algorithm is to classify a new object based on attributes and training samples [26]. The classifiers do not use any model to fit and are only based on memory [24]. The k-NN algorithm uses neighborhood classification as the prediction value of the new query instance. The traditional k-NN classification algorithm may have three limitations: (a) calculation complexity due to the usage of all training samples for classification, (b) the performance of algorithm solely depends on the training set and (c) the samples contain no apparent weight difference. The k-NN classification algorithm predicts the test sample’s category according to the k training samples which are the nearest neighbors to the test sample and determines it to that category that has the largest probability [27]. The k-NN algorithm is rooted from this assumption that being true enough for the algorithm to be useful. k-NN captures the perception of similarity (sometimes called distance, proximity or closeness), calculating the distance between points on a graph [28].

### 2.5. Random Forest

Random forest is an ensemble learning method for classification, regression and other tasks that operates by constructing a multitude of decision trees at training time and outputting the class that is the mode of the classes (classification) or mean prediction (regression) of the individual trees. Random forests correct for decision trees’ habit of overfitting to their training sets [29]. Random forest, as its name implies, consists of a large number of individual decision trees that operate as an ensemble [30]. The factors that influence memory consumption and processing time are the amount of data and the number of trees in forest. For classification, the input test sample is fed to all decision trees and then, each decision tree associates the sample to a specific class. Majority voting is performed on the outcome of all decision trees to classify the test sample. During the training process, these trees are generated using random samples from training data. To further split each node within the tree, a random number of features are used. This randomness in tree generation, along with majority voting on the outcome of all trees in the forest, adds diversification to the random forest classifier and makes it immune to overfitting [31].

### 2.6. Support Vector Machines

Support vector machines (SVM) [32] is a powerful supervised machine learning algorithm that is most widely used in classification as support vector classification (SVC) and support vector regression (SVR) applications [33]. SVMs are widely popular due to their ability to learn well with small number of parameters, their robustness against various model violations and computational efficiency compared with other methods [34]. The support vector relates to points that are closest to the hyperplane, while the margins correspond to distance between the support vectors. Support vectors are data points that are closer to the hyperplane and influence the position and orientation of the hyperplane [35]. Using these support vectors, the margins of the classifier are maximized. Deleting the support vectors will alter the position of the hyperplane, as described in Equation (2).

(2) $$h\left(x\right)=w_{0}^{T_{X}}+b_{0}$$

The SVM kernel is a function that takes low dimensional input space and transforms it to a higher dimensional space, i.e., it converts not separable problem to a separable problem. It is mostly useful in non-linear separation problems [36].

### 2.7. Decision Tree

The decision tree is a hierarchical framework made up of decision rules that splits independent variables recursively into homogenous zones. It is a decision-support approach that uses a tree-like representation of options and their potential outcomes. When making decisions, a tree-like model of decisions and their probable repercussions is used to aid in the decision process [37]. The decision tree integrates the elements in a hierarchical manner, with the most significant feature positioned at the tree’s root. Every node in the tree represents one of the characteristics and each leaf represents the most common class value. This benefits from the ability to quickly apply segmentation “rules” to elements apart from the ones that make up starting data set and the representing group is unidentified [38]. The defining “rules” of the hierarchical segmentation of elements that utilize the link between the class to which each unit belongs, and the variables identified for every unit. Each unit’s class must be known before using decision trees. The technique’s aim is to discover an optimum decision rule, for instance, a decision rule that, given a specific set of variables, permits a reliable estimate from which class every unit falls in [39].

### 2.8. LightGBM

Light gradient boosting machine (LightGBM) is a framework that utilizes gradient boosting learning and histogram-based algorithms, while it is also similar to tree-based machine learning algorithms. LightGBM uses vertical tree topology, while other similar algorithms use horizontal tree topologies. This means that LightGBM grows the tree leaf-wise (best-first) compared to level-wise for other tree-based algorithms [40]. Theoretically, leaf-wise algorithms contribute to minimizing more loss as compared to level-wise algorithms. It selects the leaf having maximum data loss to grow. Similar to any other decision tree algorithm, LightGBM is also sensitive to overfitting for smaller datasets. The finding of the optimum split points is usually the most time-consuming part in learning a decision tree. LightGBM can easily operate with large data size and utilizes lower memory while focusing on the accuracy of results [23].

## 3. Results

In this section, the characterization of the pressure in terms of pressure–voltage response, pressure–resistance relationship, senor durability and sensor drift are presented. The classification performances of the machine learning algorithms—k-NN, SVM, decision tree, random forest and LightGBM—are also discussed.

### 3.1. Characterization of Pressure Sensor

**Figure 7:**
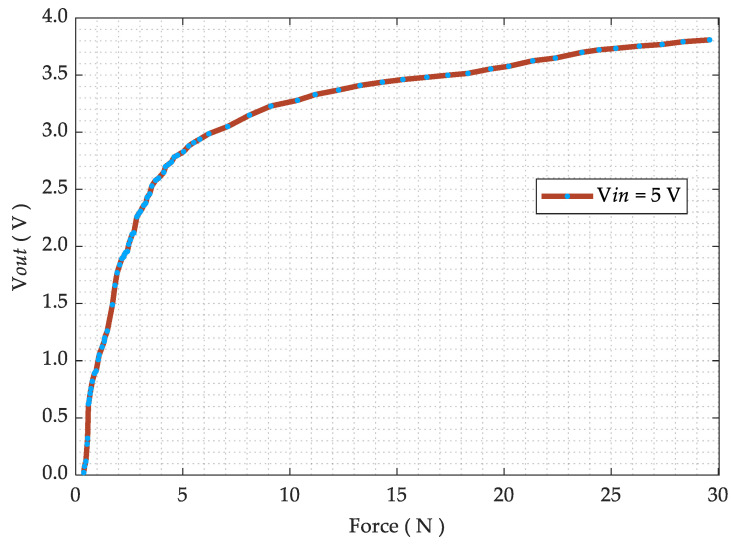
Voltage–pressure graph, when the pressure sensor is connected in a 5.0 V voltage divider configuration using a 10 kΩ 0.1% precision resistor.

The pressure–voltage relationship is obtained using a 5.0 V voltage divider configuration utilizing a high precision divider resistor of 10 kΩ. Voltage readings are acquired using a digital multimeter (Rhode and Schwarz HMC-8012) and the applied force using a dynamometer (Lutron FG-6020SD) mounted on a manual horizontal translation stage. The acquired voltage–pressure graph is then presented in Figure 7.

**Figure 8:**
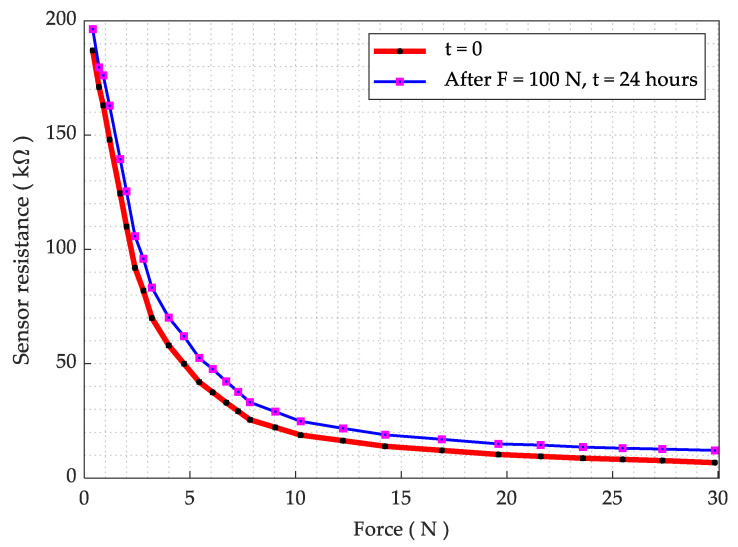
Resistance as a function of applied force.

The resistance data as a function of applied force are obtained by employing a similar experimental setup as for pressure–voltage test without voltage divider. A rubber-based actuator (30 Shore D) has been attached to a dynamometer. The force sensitivity range is measured as ~0.2 to 30 N and the activation force is ~0.2 N in this sensor design. The test is performed at room temperature and the resulting curve is presented in Figure 8.

**Figure 9:**
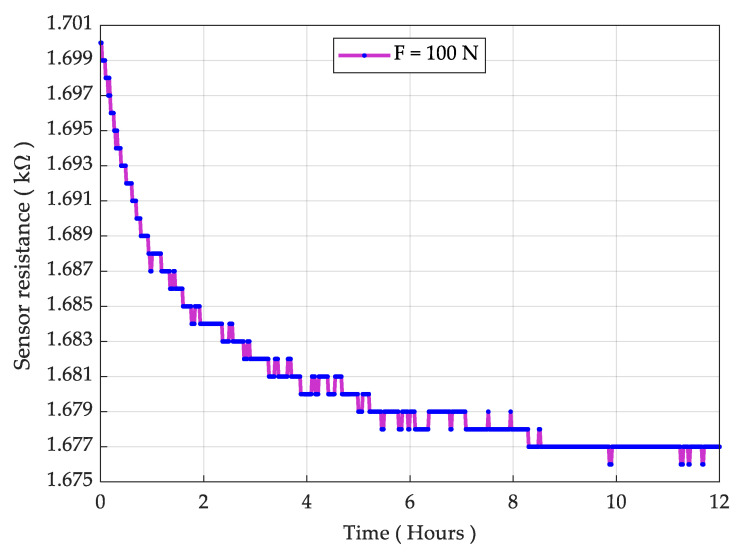
Sensor drift under the load of 100 N downwards force (10 kg).

For the durability test, we put a sensing element under 100 N force (about 3.3 times the normal range) for a period of 24 h, utilizing a precision motorized force tester (Mecmesin MultiTest 2.5-*dV*). The resistance is logged using a UT61D multimeter. The resistance as a function of force is measured once again after the durability test and the result is presented in Figure 8. The result showed a slight increase in the resistance; however, the overall sensing behavior remained nearly unchanged. Then, we considered the sensor for physical and mechanical deformation and it was realized that the sensor PET sheet does not deform physically at 100 N (10 Kg) load. The result for the sensor drift is shown in Figure 9, while the measured drift was 1.41% at 100 N over 24 h. It is also noted that a pressure in excess of 200 N/cm^2^ causes irreversible damage to the sensor.

### 3.2. Performance of Machine Learning Algorithms

In order to evaluate the performance and advantages of each step in the sitting posture monitoring system, the results of the classification algorithms are detailed in terms of classification behavior and required system time to compile the algorithm. This will also serve as a reference to evaluate the overall performance of the system. The classifiers are trained using the training data that comprise four different sitting postures, as mentioned in previous section. The test data are then used to determine the average classification accuracy of each classifier. The classifiers are trained without any dimensionality reduction. The key parameters of the classification algorithms are summarized in Table 2.

The data are in numeric form, processed by the microcontroller’s ADCs from all 16 sensing elements. The dataset is composed of four categorical classes (right-lean, left-lean, forward-lean and backward-lean in the dataset). There are 16 columns of numerical data, so there are 16 independent variables and the total number of observations are 256, whereas there are a total of 4096 data points. For training purposes, all 16 ‘sensors’ data are used; hence, the dimensionality reduction methods are not utilized. The train–test data splitting is carried out using the (train_test_split) function and the train size is 0.8, or 80%, while test size is 0.2, or 20%. The random state is not set for any of the mentioned machine learning algorithm; thus, the test data would be randomized in each run.

**Figure 10:**
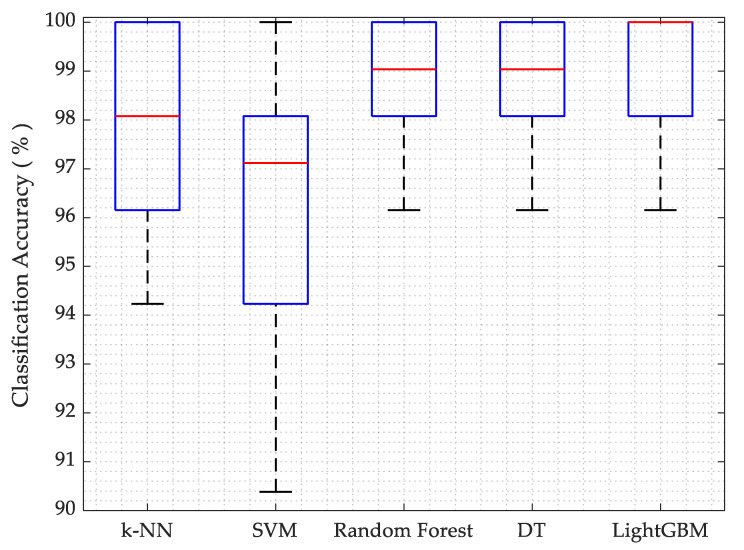
Classification accuracy for machine learning algorithms.

For k-NN, default settings from scikit-learn are used. However, the number of neighbors are set to 3 in our implementation (n_neighbors = 3). It is also noted that the classification accuracy is also similar when (n_neighbors = 5) is used. The k-NN algorithm provided sufficiently better average accuracy for posture classification and less compile time. While using the SVM algorithm, the default settings from scikit-learn are used and (gamma = ‘scale’) is employed. SVM illustrates the worst (least accuracy) among the classifiers due to its binary classification tendency; however, the compile time is sufficiently short. In random forest, default settings from scikit-learn are used that have the required estimators (n_estimators = 100). Random forest yielded very good average classification accuracy: up to 98.65%. Random forest requires high processing resources because of its complexity and takes a longer time to compile, making it not very suitable for use on the raspberry pi 3B. While employing the decision tree algorithm, the default settings from scikit-learn and criterion = ‘gini’ are used. The decision tree also resulted in a better average accuracy and short compile time. For LightGBM, the default settings are used and n_estimators = 100 are already set in the settings. Light GBM performed the best, although it took relatively more compile time than the others; however, it is still faster than the random forest. The average accuracies from the five classifiers are shown as a boxplot in Figure 10.

## 4. Standalone System

**Figure 11:**
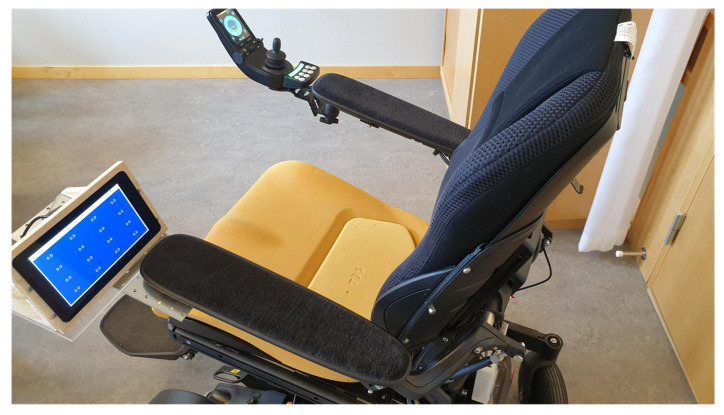
Standalone embedded system mounted on a wheelchair.

A raspberry pi 3B using the Raspbian OS constructs the core of the portable information and monitoring system. The incoming data are processed in Spyder IDE using Python programming language and relevant information is, hence, displayed on the 7-inch touch display, as shown in Figure 11.

In the standalone monitoring system, the pressure data coming from read-out electronics are received through serial communication over USB on raspberry pi 3B. As the standalone embedded system is capable of internet connectivity through onboard Wi-Fi, the data can be easily sent and stored on a web database, cloud or a network attached storage (NAS) system for the evaluation of daily sitting routines and long-term evaluation of sitting behavior. Different IDEs for Python can be used to run the posture recognition algorithms. The average compile time for popular Python IDEs for raspberry pi is compared in Table 3. To evaluate the compile time of employed machine learning algorithms for a standalone embedded system, raspberry pi 3B and raspberry pi 4B were used.

The proposed standalone embedded system was also compared with a standard desktop PC (processor: Intel Core i7, RAM 32 Gb, 256 Gb SSD storage); as naturally expected, the standard PC surpassed the standalone embedded system in terms of computing time, evaluated using Spyder IDE. The average accuracy and average compile time were compared for different machine learning algorithms between a standard PC and a standalone embedded system and the results are listed in Table 4.

**Figure 12:**
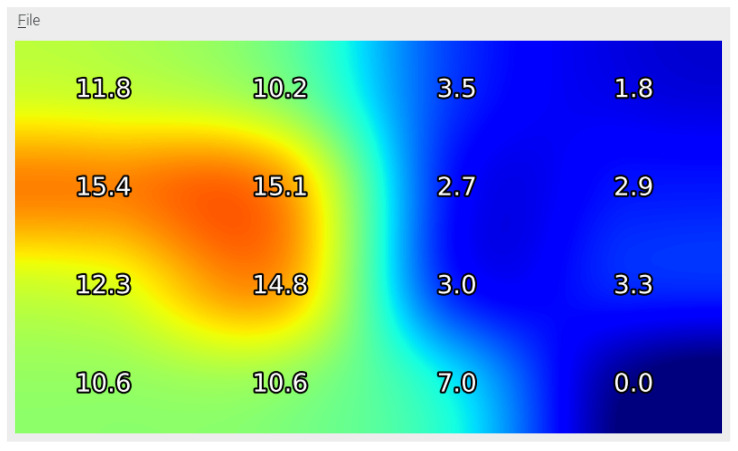
A screenshot of pressure map showing pressure values (in kPa) on proposed embedded system’s screen when seated person leaned to left.

An interactive pressure map shows the current pressure exerted by a sitting person in real-time on the raspberry pi screen, as shown in Figure 12. The volunteer in this test was a male, had a weight of 95 kg and a heavy-boned physique. The pressure map illustrates the pressure intensity of all sixteen unit elements of the sensor array. The pressure intensity is shown with RGB color space. It also shows numerical values of current pressure with respect to sitting orientation. The values are updated every one second on the screen. The real-time animated pressure map is drawn on-screen using PyQt5 framework and the updating frequency is 5 Hz.

**Figure 13:**
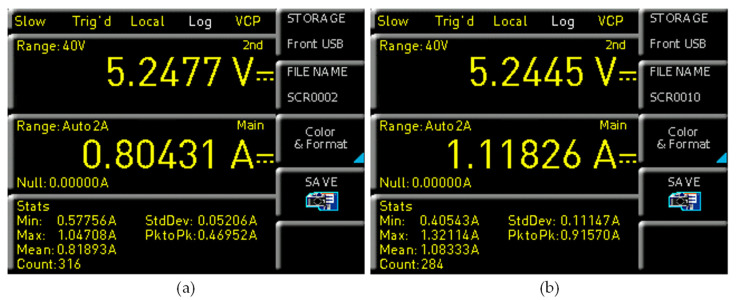
Electrical current consumption of standalone embedded system (**a**) Raspberry pi 3B; (**b**) Raspberry pi 4B.

The average power consumption for the developed embedded system, including touch display and read-out electronics (based on a mean value of current), w 4.28 W (818 mA and 5 V) for raspberry pi 3B, whereas raspberry pi 4B consumes 5.68 W (1083 mA and 5 V), as shown in Figure 13. The readings for the current consumption and voltages were carried out using a digital multimeter (Rhode and Schwarz HMC-8012) by employing the standard electrical methods.

## 5. Discussion and Conclusions

This research presented the layout of a standalone embedded system comprising five classification algorithms that are capable of identifying sitting posture abnormalities on a wheelchair with a prediction performance of up to 99.03%. As a result of the pressure data that are processed by machine learning algorithms, our system is able to classify four leaning positions by means of some effective classification algorithms using machine learning (for instance, k-NN, SVM, decision tree, random forest and LightGBM, as discussed in the previous section). The results from k-NN, SVM and decision tree show the possibility to implement these algorithms in real-time posture recognition schemes, as the processing is efficient and easy in terms of processing speed and resource consumption. All of the classification algorithms that are employed in this work resulted in a satisfactory accuracy. LightGBM offers better accuracy than the others, but it requires a slightly larger compile time; however, it is still faster than the random forest algorithm.

The use of machine learning in sitting posture recognition for wheelchair users improves the recognition accuracy from the obtained pressure data. Recognizing an improper sitting posture is easily feasible and the user would be advised accordingly. The sitting posture recognition system offers an effective way for rehabilitation and pressure ulcer prevention for wheelchair users and it is equally beneficial for able-bodied users. The standalone embedded system for posture recognition and information was developed that can be easily mounted on any wheelchair (powered or non-powered) to show information related to sitting posture. The raspberry pi 3B and raspberry pi 4B are capable of performing the pressure distribution monitoring task easily; nonetheless, the run-time classification might be difficult for the raspberry pi 3B, as it requires a longer compile time to use machine learning algorithms. In addition, raspberry pi 4B carry out tasks more quickly; however, it will consume a bit more power in doing so.

## 6. Future Work

The pressure data collection stage can be improved in order to further enhance the overall classification accuracy of the system. Pressure data from more volunteers can be added to the dataset to improve its accuracy. Furthermore, the proposed algorithms could also be utilized efficiently in post-processing of the data, both on the raspberry pi and on a standard PC. In future works, some other predictor data variables, such as age, height and weight, can be used to develop more precise classification algorithms. Currently, the pressure monitoring program is running manually on the embedded monitoring system; however, it could be made to run at the startup of the raspberry pi system. The on-screen menu and user interface can be modified and enhanced to be made even more interactive for users.

## Figures and Tables

**Table 1:** Volunteers participated in data set compilation.

Participants	Participants Count	Weight Range (kg)	Average Weight (kg)
Male	25	57–132	77
Female	7	49–88	62

**Table 2:** Machine learning algorithms’ functional parameters.

No	Classifier	Parameters
1	k-nearest neighbors (k-NN)	n_neighbors = 3, leaf_size = 30, metric = ‘minkowski’
2	Random Forest (RF)	n_estimators = 100, criterion = ‘gini’, min_samples_split = 2, min_samples_leaf = 1
3	Support Vector Machines (SVM)	SVM type: SVC, degree = 3, C = 1.0, kernel = ‘rbf’, gamma = ‘scale’, random_state = None
4	Decision Tree (DT)	Criterion = ‘gini’, min_samples_split = 2, min_samples_leaf = 1
5	Light Gradient Boosting Machine (LightGBM)	n_estimators = 100, boosting_type = ‘gbdt’, num_leaves = 31, max_depth = −1, learning_rate = 0.1, subsample_for_bin = 200,000

**Table 3:** Average compile time on popular Python IDEs.

IDE/Version	Algorithm	Raspberry Pi 3B Average Compile Time (s)	Raspberry Pi 4B Average Compile Time (s)
Spyder 3.3.3	k-NN	0.951	0.452
	Random forest	2.181	0.942
	SVM (linear)	0.904	0.423
	Decision Tree	0.812	0.394
	Light GBM	1.576	0.638
Thonny 3.3.1	k-NN	8.305	3.182
	Random forest	12.65	3.936
	SVM (linear)	8.734	3.214
	Decision Tree	8.556	3.375
	Light GBM	9.956	4.526
Geany 1.33	k-NN	5.805	2.754
	Random forest	7.812	3.245
	SVM (linear)	6.154	2.702
	Decision Tree	6.632	2.762
	Light GBM	6.305	2.824

**Table 4:** Average accuracy and compile time on Spyder IDE (Python 3.7.3).

Processor/RAM	Algorithm	Average Accuracy %	Average Compile Time (s)
Desktop CPU Core i7 Intel 4790K (4.0 GHz) 32 GB DDR3-1600 MHz	k-NN	98.07	0.054
	Random forest	98.65	0.167
	SVM (linear)	95.96	0.051
	Decision Tree	98.85	0.049
	Light GBM (64-bit)	99.03	0.131
Raspberry Pi 3B BCM2837 (1.2 GHz) 1 GB LPDDR2-900 MHz	k-NN	97.88	0.951
	Random forest	98.46	2.181
	SVM (linear)	95.00	0.904
	Decision Tree	97.11	0.812
	Light GBM (32-bit)	99.03	1.576
Raspberry Pi 4B BCM2711 (1.5 GHz) 4 GB LPDDR4-3200 MHz	k-NN	98.27	0.452
	Random forest	98.07	0.942
	SVM (linear)	95.00	0.423
	Decision Tree	97.81	0.394
	Light GBM (32-bit)	99.03	0.638

## Data Availability

Not applicable.
